# Supplementary material for: A set of multi-entry identification keys to African frugivorous flies (Diptera, Tephritidae)
Source: Zookeys. 2014 Jul 24;(428):97–108. doi: 10.3897/zookeys.428.7366 (PMC4143993; doi:10.3897/zookeys.428.7366)
Supplement: Supplementary material 6 — Key to Ceratitis [file zookeys-428-097-s006.zip › SF6_ZooKeys_key to Ceratitis/key/SF6_ZooKeys_key to Ceratitis/Media/Html/Ceratitis anonae .htm]

Ceratitis (Pterandrus) anonae Graham


***Ceratitis*** **(*Pterandrus*) *anonae* Graham**

*Ceratitis* *anonae* Graham, 1908: 114.

Body
length: 5.06 (4.35-5.90) mm; wing length: 5.15 (4.45-5.75) mm.

##### Male

##### Head: Antenna yellow. First flagellomere three times as long as pedicel. Arista with short to moderately long rays; ventral rays shorter and sparser than dorsal rays, especially basally. Frons pale, sometimes completely yellow, center yellow; with short scattered setulae distinctly darker than frons. Frontal setae well developed. Face white, sometimes yellowish white. Genal seta and setulae dark, well developed.

Thorax:
Postpronotal lobe white, sometimes yellowish white; without spot. Scutal
pattern: ground color ash-gray; with streaks and darker markings but without
distinct spots or clearly defined stripes except prescutellar white markings
separate, sometimes with pale area in between. Scapular setae dark. Scutellum
white, sometimes yellowish white, basally without spots, apically with three
separate black spots, extending to basal 0.33, sometimes only to basal
half. This has a strage meaning. The measurment should relate to apical 0.66 or
0.5 (global) I am not sure, the spots are apical spots reaching ‘forwards’
towards the basal margin. In some species they will reach the basal margin of
the scutellum, in others only till halfway (‘basal half’ or ‘apical half’,
whatever you choose),  still in others till basal third. Referring this forward
extension in relation to the apical length, sound a bit strange to me. I would
therefore prefer to keep it in relation to the basal margin. However, if you
feel very strongly about the apical aspect, you can still do a global change as
such. Anepisternum on ventral 0.33-0.5 brown; setulae pale.

Legs:
Yellow except where otherwise noted; setation typical for subgenus, mainly dark.
Foreleg: femur dark anteriorly along entire dorsal margin, posteriorly along
dorsal 0.66, with poorly developed bush of dispersed long dark setulae along
entire length, posterodorsal setulae longer; ventral setae dark. Midleg: femur
largely brownish black, anteriorly with silvery shine when viewed from certain
angle, only distal end pale; ventrally with dark feathering along entire
length, basally somewhat less dense; tibia broadened; largely brownish black
with silvery shine when viewed from certain angle, with black feathering
dorsally along distal 0.9 and ventrally along distal 0.8. Hindleg: femur dark
brown except distally, at apical 0.25 with longer setulae dorsally and
ventrally.

Wing:
Markings yellowish brown. Interruption between marginal and discal bands near
vein R_1_ clear and complete; discal band often partly or completely
interrupted in discal cell; cubital band free; medial band absent; crossvein
R-M opposite middle of discal cell. Apex of vein R_1_ distal to level
of crossvein R-M. Crossvein DM-Cu oblique anterobasally.

Abdomen:
Mostly yellow. Border between tergite 1 and 2 narrowly black. Tergites 2 and 4
with pale gray band occupying almost entire tergite, at most narrowly yellow
anteriorly. Tergite 3 with distinct brownish black band along posterior half;
rarely interrupted medially. Tergite 5 with basal 0.33 brownish, usually
divided medially into two spots; posteriorly narrowly brownish. Male epandrium
in lateral view with lateral surstylus curved, posterior lobe short.

Female

As
male except following characters. Anepisternal pilosity on ventral 0.33 partly
dark, especially centrally. Legs without feathering; femora
yellow, forefemur posteriorly, and midfemur and hindfemur anteriorly on basal
0.66 often with brownish streaks; sometimes femora completely yellowish brown;
forefemur posteroventrally with dark pilosity. Wing with discal band complete.
Oviscape shorter than preabdomen. Aculeus at most six times as long as wide;
tip with distinct apical indentation and lateral margin slightly sinuous.

(Description
after De Meyer & Freidberg, 2006)
